# Prophylactic Ghosh Uterine Compression Suture Technique to Prevent Severe Postpartum Hemorrhage in Placenta Accreta

**DOI:** 10.7759/cureus.109554

**Published:** 2026-05-24

**Authors:** Sharda B Ghosh, Fadia AlKhalil, Shadi Alkhalil

**Affiliations:** 1 Obstetrics and Gynaecology, Al Zahra Hospital Dubai, Dubai, ARE; 2 Obstetrics and Gynaecology, Saudi German Hospital, Dubai, ARE

**Keywords:** alternate sequential suture tightening technique (asstt), compression suture, placenta accreta, postpartum hemorrhage, uterus

## Abstract

Placenta accreta is associated with severe postpartum hemorrhage (PPH) and may require massive transfusion and emergency hysterectomy, contributing to substantial maternal morbidity and mortality. The prophylactic use of the Ghosh uterine compression suture (Alternate Sequential Suture Tightening Technique (ASSTT)) may reduce hemorrhagic complications. This report describes two patients (one primigravida and one gravida 2) with antenatally diagnosed placenta accreta based on ultrasound and magnetic resonance imaging (MRI), without other comorbidities, who underwent elective-term cesarean delivery with prophylactic ASSTT and circumferential cervical suturing. The mean time to place the Ghosh suture was four to five minutes, and the mean estimated blood loss was 450 mL (range: 450-500 mL). Neither patient required a transfusion, and both were discharged on postoperative day 3 without complications. After lactation, both resumed regular menses and subsequently achieved uncomplicated pregnancies. The first patient conceived spontaneously three years later and underwent an uncomplicated term repeat cesarean delivery. The second patient conceived via in vitro fertilization (IVF) for sex selection 3.5 years later; antenatal imaging was unremarkable. However, a morbidly adherent placenta was identified intraoperatively, and ASSTT with circumferential cervical suturing was repeated. The patient was discharged on postoperative day 3 without complications. These cases suggest that prophylactic ASSTT, combined with circumferential cervical suturing, may be a feasible uterus-sparing strategy in selected patients with placenta accreta.

## Introduction

The reported incidence of placenta accreta has increased in recent years, largely in parallel with rising rates of abnormal placentation and uterine instrumentation, including cesarean delivery and prior placenta accreta [1]. Clinical outcomes are strongly influenced by timely antenatal diagnosis, surgical experience, multidisciplinary planning, and access to appropriate resources [1,2]. Delayed or missed diagnosis may lead to severe postpartum hemorrhage (PPH), massive transfusion, and emergency hysterectomy in approximately 60-70% of cases [3,4]. The B-Lynch uterine compression suture was introduced as a surgical option for uncontrolled hemorrhage due to uterine atony [4-6]. Subsequent reports of complications prompted the development of modified compression suture techniques, including those described by Hayman [7], Cho [8], Pereira [9], Ouahba [10], Hackethal [11], and Matsubara [12]. In 2015, a modified Hayman approach, the Alternate Sequential Suture Tightening Technique (ASSTT; Ghosh suture), was reported by the corresponding author, following successful use in 12 cases of severe, uncontrolled PPH [13]. The overarching goal of uterine compression sutures is to reduce maternal morbidity and mortality while preserving fertility. Although no single technique is universally superior, a method that is simple, rapid, effective, and associated with minimal complications is clinically valuable [13]. This report describes outcomes of the Ghosh suture combined with circumferential cervical suturing in two cases of placenta accreta.

## Case presentation

Case 1

**Figure 1:**
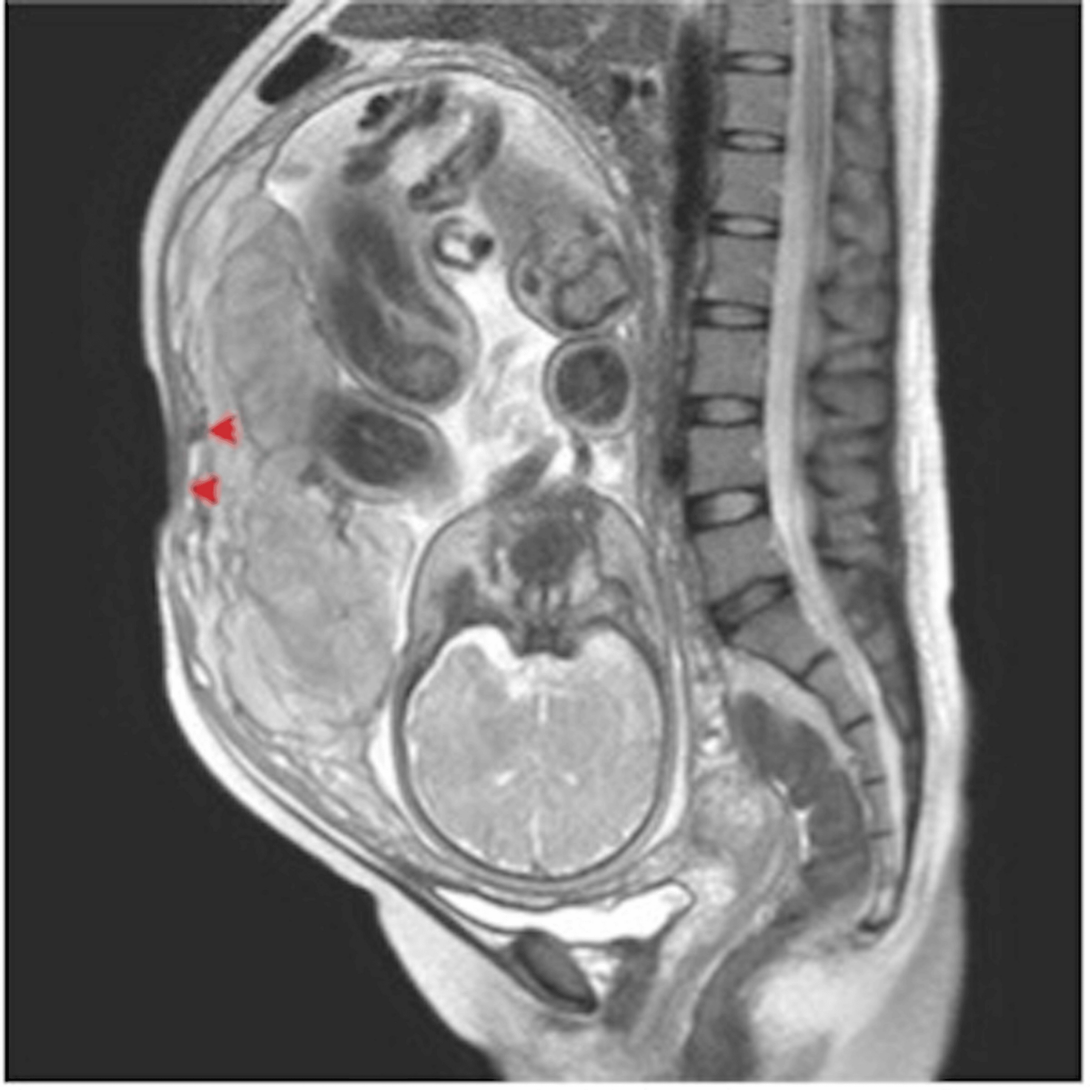
MRI showing placenta accreta (red arrowheads) with a fetus (Case 1).

A primigravida conceived spontaneously and had undergone laparoscopic myomectomy six months before conception. Antenatal screening, including hemoglobin, was within normal limits. Placenta accreta was diagnosed at the second-trimester anomaly scan and confirmed by magnetic resonance imaging (MRI) in the third trimester (Figure 1). An elective lower-segment cesarean section (LSCS) was performed at term. After delivery and before placental removal, the Ghosh uterine compression suture (ASSTT) was placed. The placenta was adherent and was removed with the superficial endometrium, after which the ASSTT sutures were tightened. Oxytocin 5 IU was administered as an intravenous bolus, followed by an infusion containing 40 IU. A circumferential cervical suture was then placed to reduce bleeding from anastomosing vaginal arteries. The combined suturing procedures were completed in five minutes. The estimated blood loss was 500 mL, and no blood transfusion was required. The patient was discharged on postoperative day 3. Menses resumed after lactation without abnormalities. Three years later, she conceived spontaneously and underwent an uncomplicated elective term repeat cesarean delivery.

Case 2

**Figure 2:**
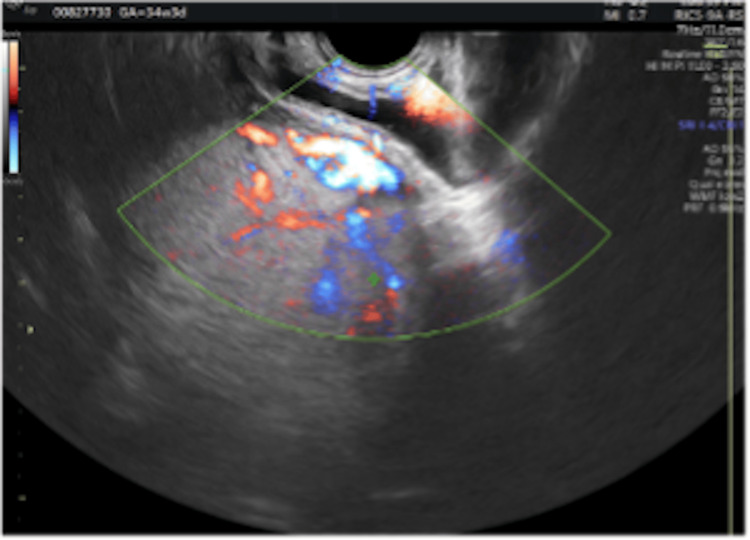
Ultrasound showing placenta accreta (Case 2).

**Figure 3:**
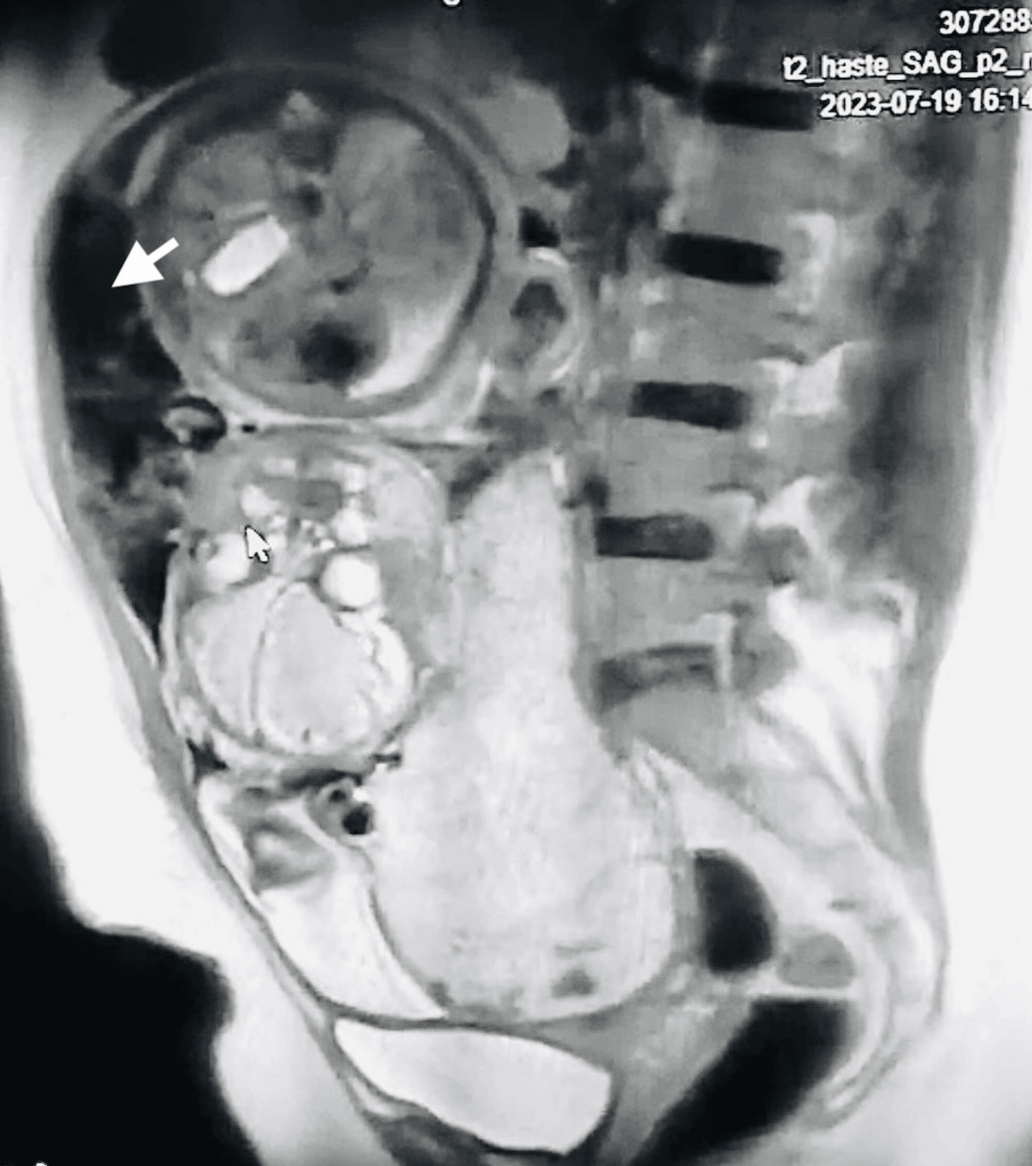
MRI showing placenta accrete with large white arrow (Case 2).

A gravida 2 with a prior cesarean delivery was diagnosed with placenta accreta in the second and third trimesters based on ultrasonography (Figure 2) and MRI findings (Figure 3). Antenatal screening investigations, including routine blood tests, were within normal limits. An elective cesarean delivery was performed at term. After delivery and before placental removal, the Ghosh uterine compression suture (ASSTT) was placed. The placenta was removed, and the ASSTT sutures were tightened immediately thereafter. A circumferential cervical suture was applied to reduce bleeding from anastomosing vaginal arteries. The combined suturing procedures were completed in five minutes. The estimated intraoperative blood loss was 400 mL. No blood transfusion was required, and the patient was discharged on postoperative day 3. Menses resumed after lactation without abnormalities. Three and a half years later, the patient conceived via in vitro fertilization (IVF) for sex selection. Antenatal imaging was reported as normal. At repeat cesarean delivery, the placenta was again morbidly adherent; therefore, ASSTT and a circumferential cervical suture were reapplied. Oxytocin 5 IU was administered as an intravenous bolus, followed by an infusion containing 40 IU. Estimated blood loss was 450 mL. The patient did not require a transfusion and was discharged on postoperative day 3.

Alternate Sequential Suture Tightening Technique (Ghosh uterine compression suture)

Position and Technique

After induction of anesthesia, the patient is positioned, prepped, and draped under sterile conditions for an LSCS [13].

Steps for the Prevention and Management of Intrapartum PPH

After delivery, suspected atonic PPH is initially managed with uterine massage and appropriate medical therapy [13]. If bleeding persists, prompt resuscitation is initiated, and blood products are arranged and transfused as required [13]. When medical therapy fails, the uterus is exteriorized, and bimanual compression is used to assess whether the ASSTT (Ghosh suture) is likely to be effective [13].

Prophylactic Use of the Ghosh Uterine Compression Suture (ASSTT) in Placenta Accreta/PPH

After delivery, the uterus is exteriorized, and ASSTT is applied prophylactically to prevent or control hemorrhage associated with placenta accreta and PPH [13].

Steps of the Ghosh Uterine Compression Suture [13]

**Figure 4:**
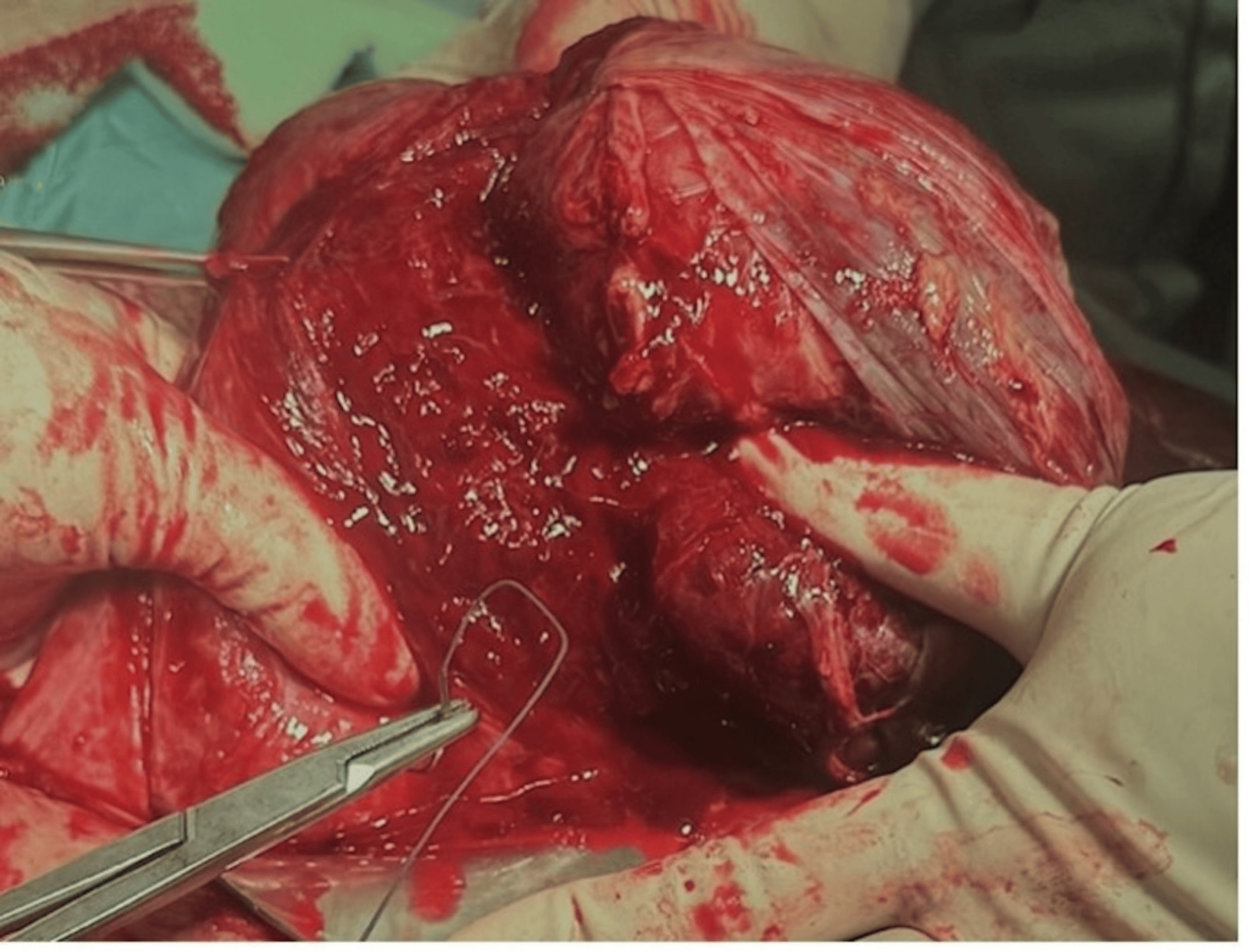
Taking the ASSTT suture (Case 2). ASSTT: Alternate Sequential Suture Tightening Technique

**Figure 5:**
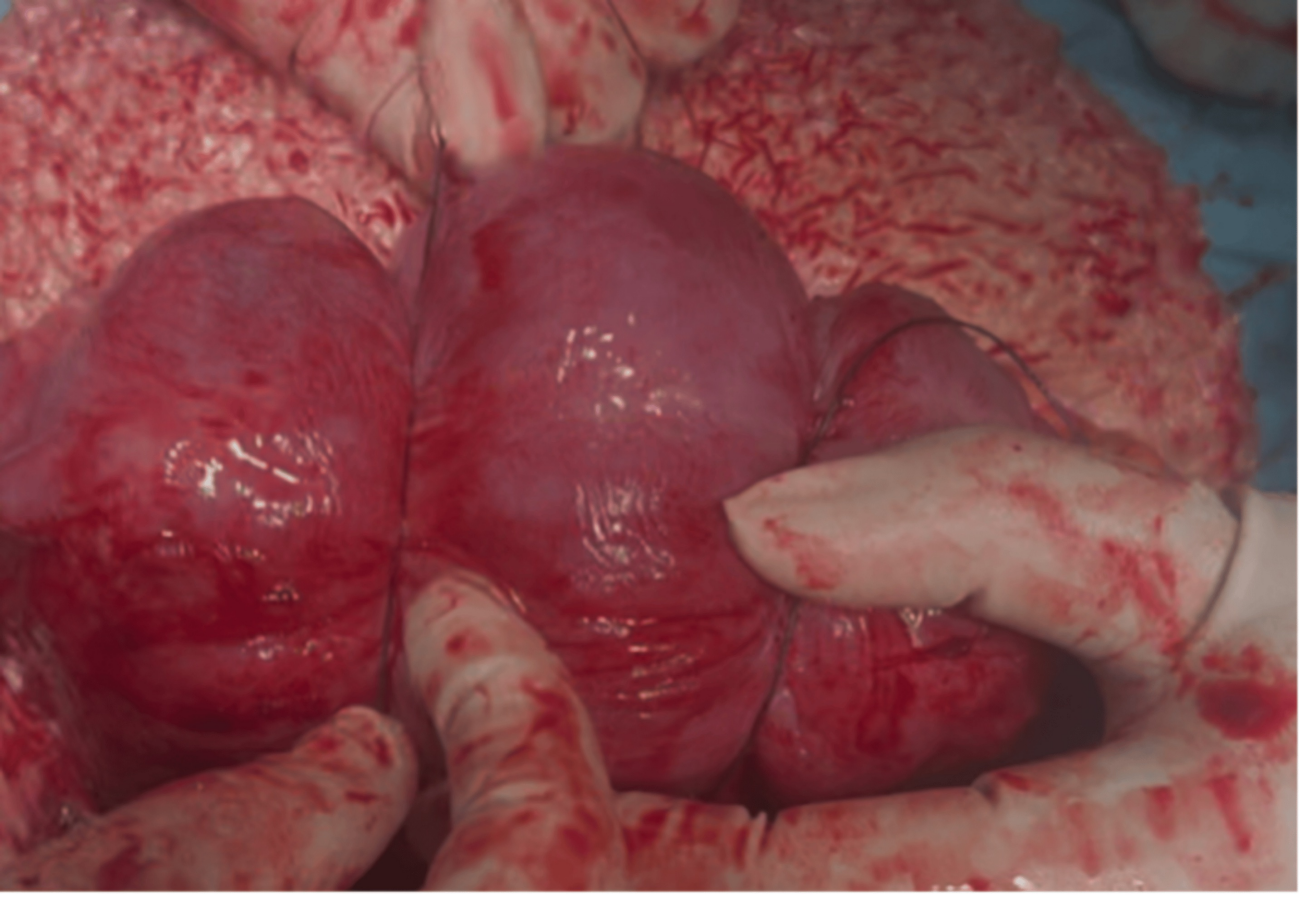
Right and left ASSTT sutures have been placed (Case 2). ASSTT: Alternate Sequential Suture Tightening Technique

**Figure 6:**
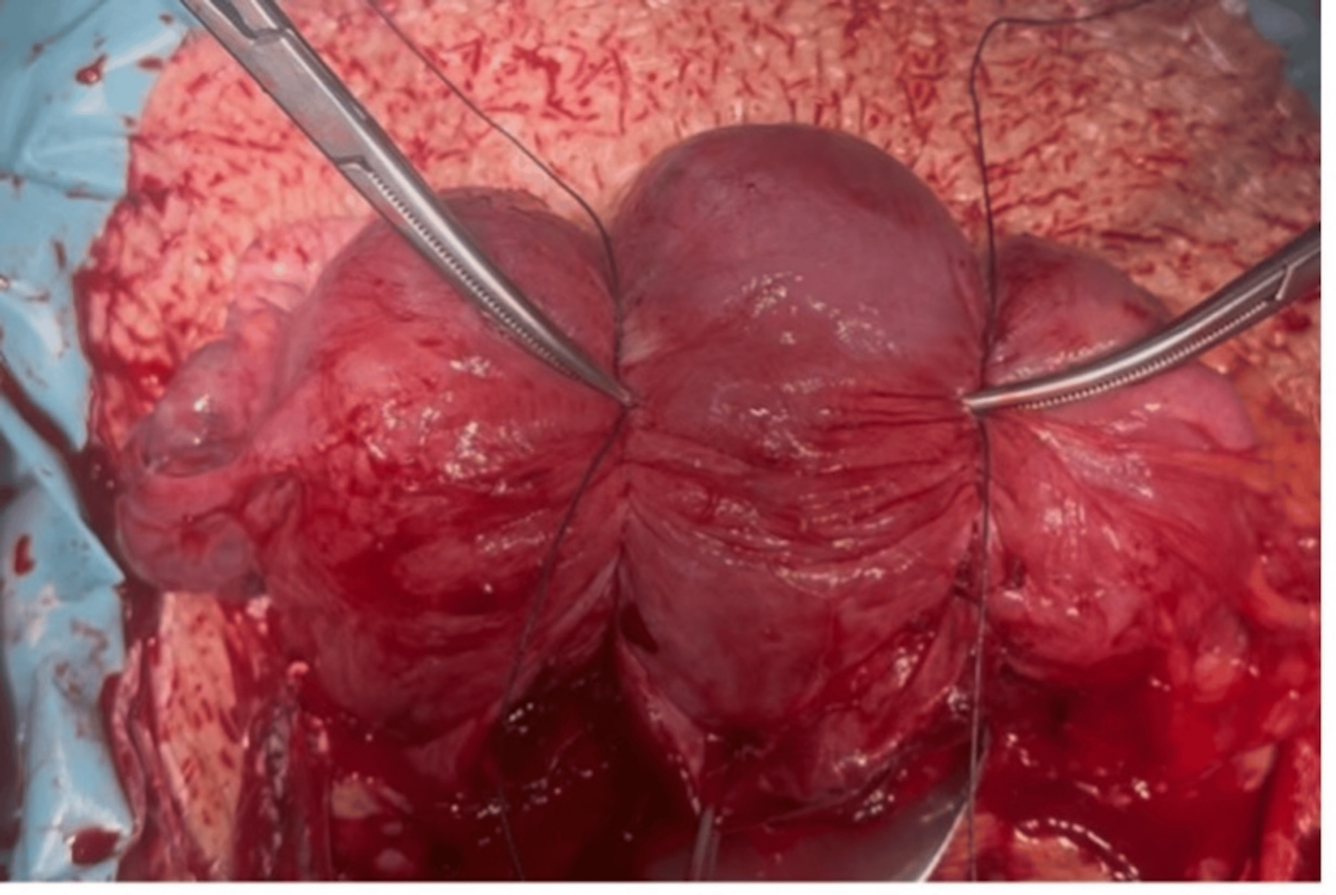
Both sutures are stepped at the knot on the fundus (Case 2).

**Figure 7:**
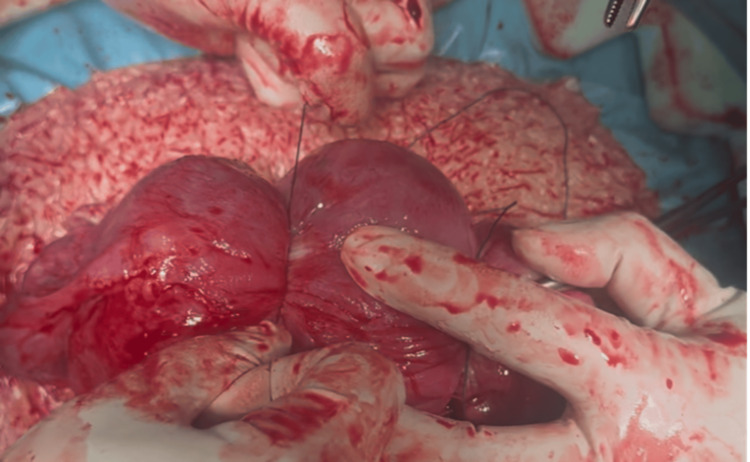
Right ASSTT suture being tightened (Case 2). ASSTT: Alternate Sequential Suture Tightening Technique

**Figure 8:**
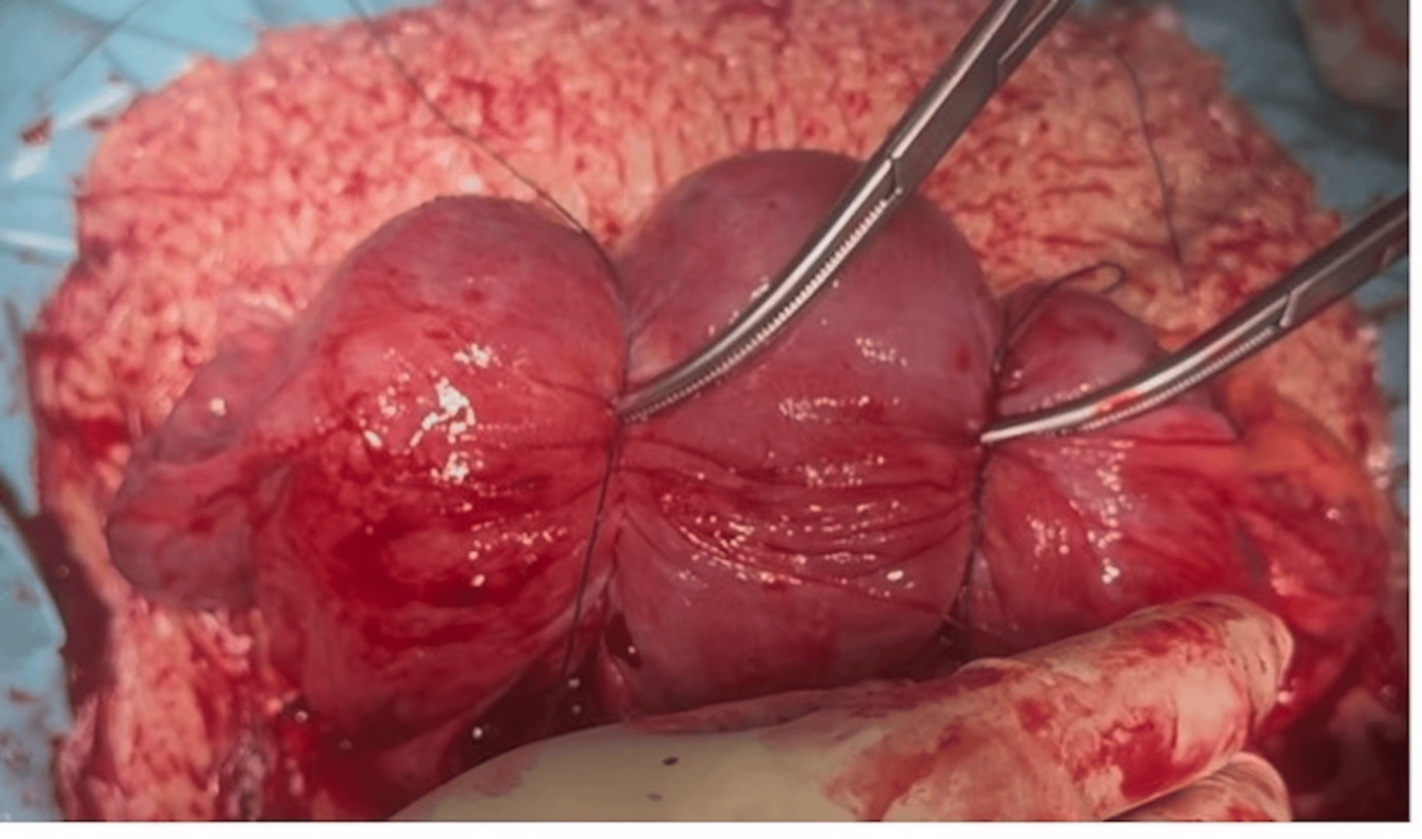
Holding the right ASSTT suture after being tightened (Case 2). ASSTT: Alternate Sequential Suture Tightening Technique

**Figure 9:**
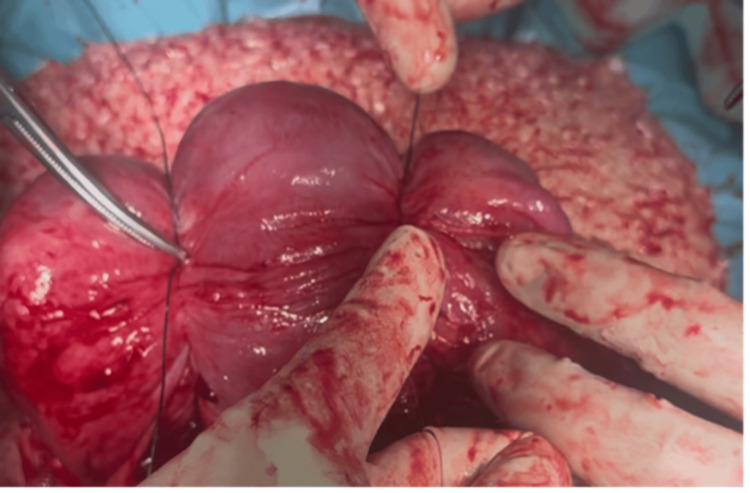
Left ASSTT suture being tightened (Case 2). ASSTT: Alternate Sequential Suture Tightening Technique

**Figure 10:**
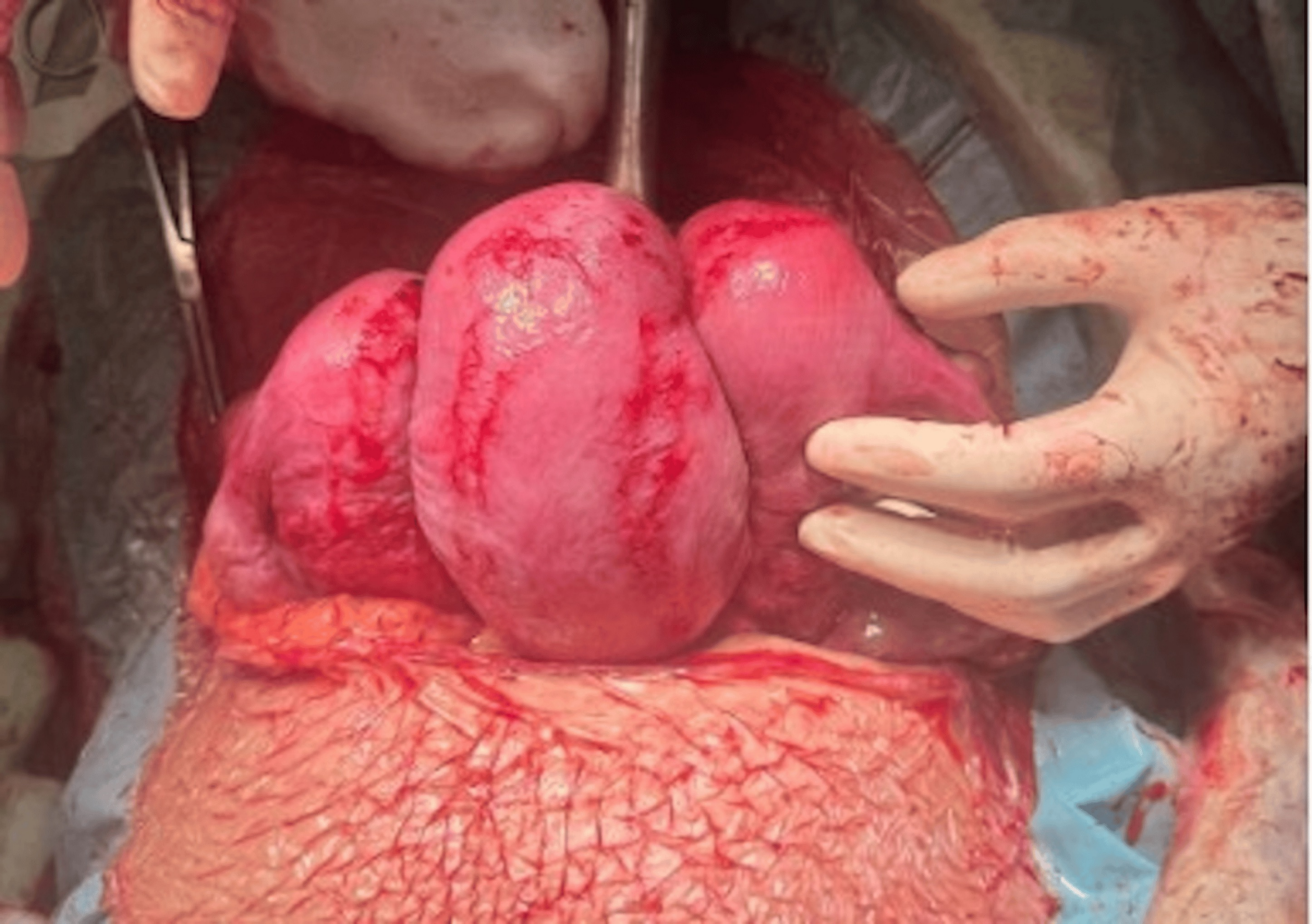
Posterior view of the well-compressed uterus with the ASSTT suture (Case 2). ASSTT: Alternate Sequential Suture Tightening Technique

Two no. 2 Vicryl sutures on straightened needles are used. On the right side, the suture is inserted through the anterior uterine wall approximately 1-2 cm above the bladder reflection, 1-2 cm medial to the lateral border of the lower uterine segment, and 1-3 cm below the lower uterine incision (Case 2, Figure 4). The needle is passed through the uterine cavity and brought out through the posterior wall at the corresponding level. The free ends are tied over the uterine fundus using a double-throw knot (Case 2, Figure 5) and temporarily secured with an artery forceps (Case 2, Figure 6). An identical suture is placed on the left side and similarly secured (Case 2, Figure 6). The right artery forceps is released, the suture is tightened further, and the suture is reclamped at the new tension point (Case 2, Figure 7). The maneuver is repeated on the left side (Case 2, Figures 8-9). Alternate sequential tightening is repeated approximately three to four times until no further tightening is possible and bleeding is controlled (Case 2, Figure 9). Each suture can typically be tightened an additional 3-5 cm beyond the initial tension. After satisfactory compression and hemostasis are achieved, the sutures are secured with square knots on both sides. The uterine incision is inspected; if hemostasis is confirmed, the uterus is closed in the standard manner (Case 2, Figures 10).

Additional Prophylactic Step for Placenta Accreta Spectrum (Circumferential Cervical Suture)

**Figure 11:**
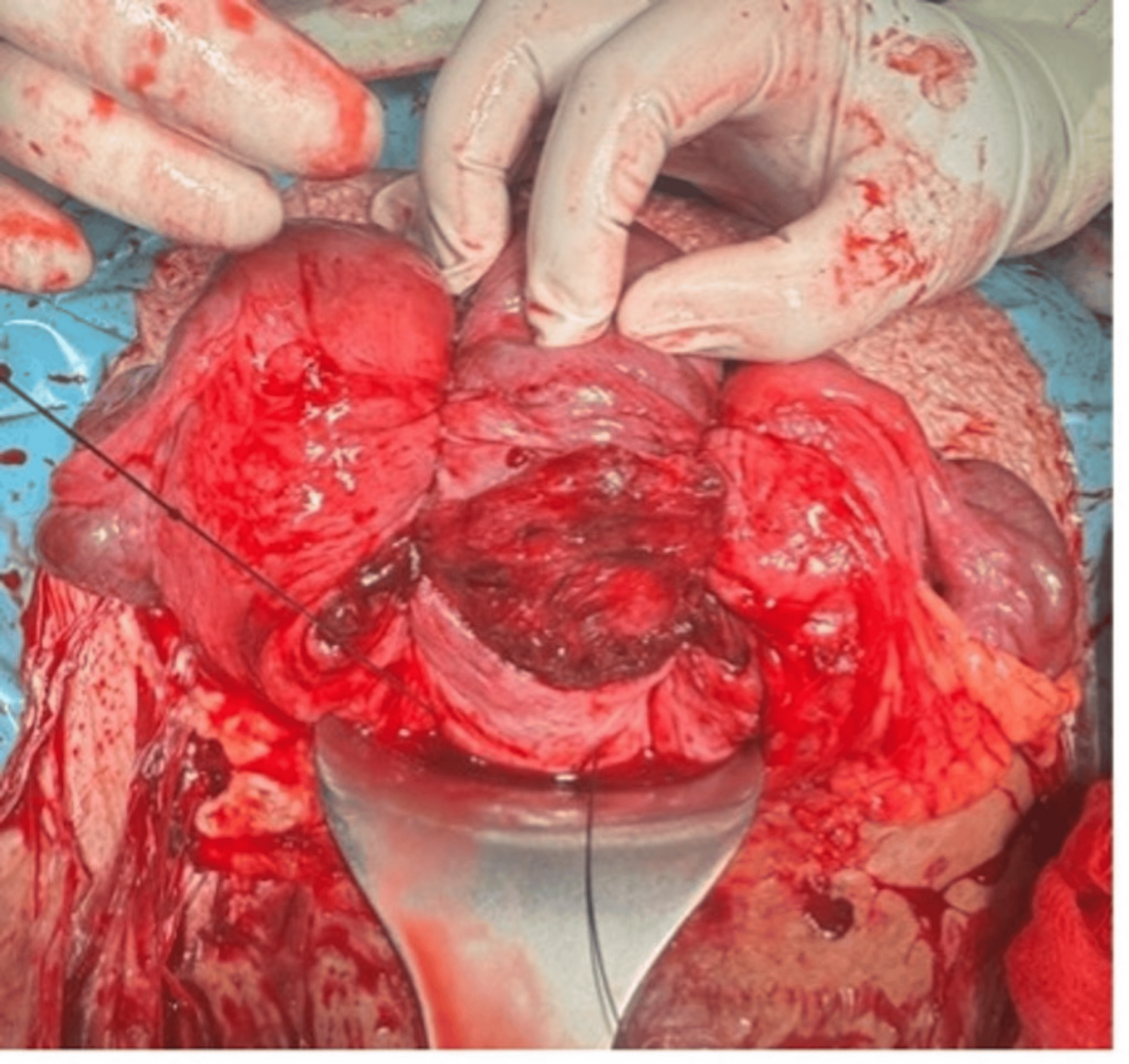
Putting circumferential cervical sutures for placenta accreta (Case 2).

**Figure 12:**
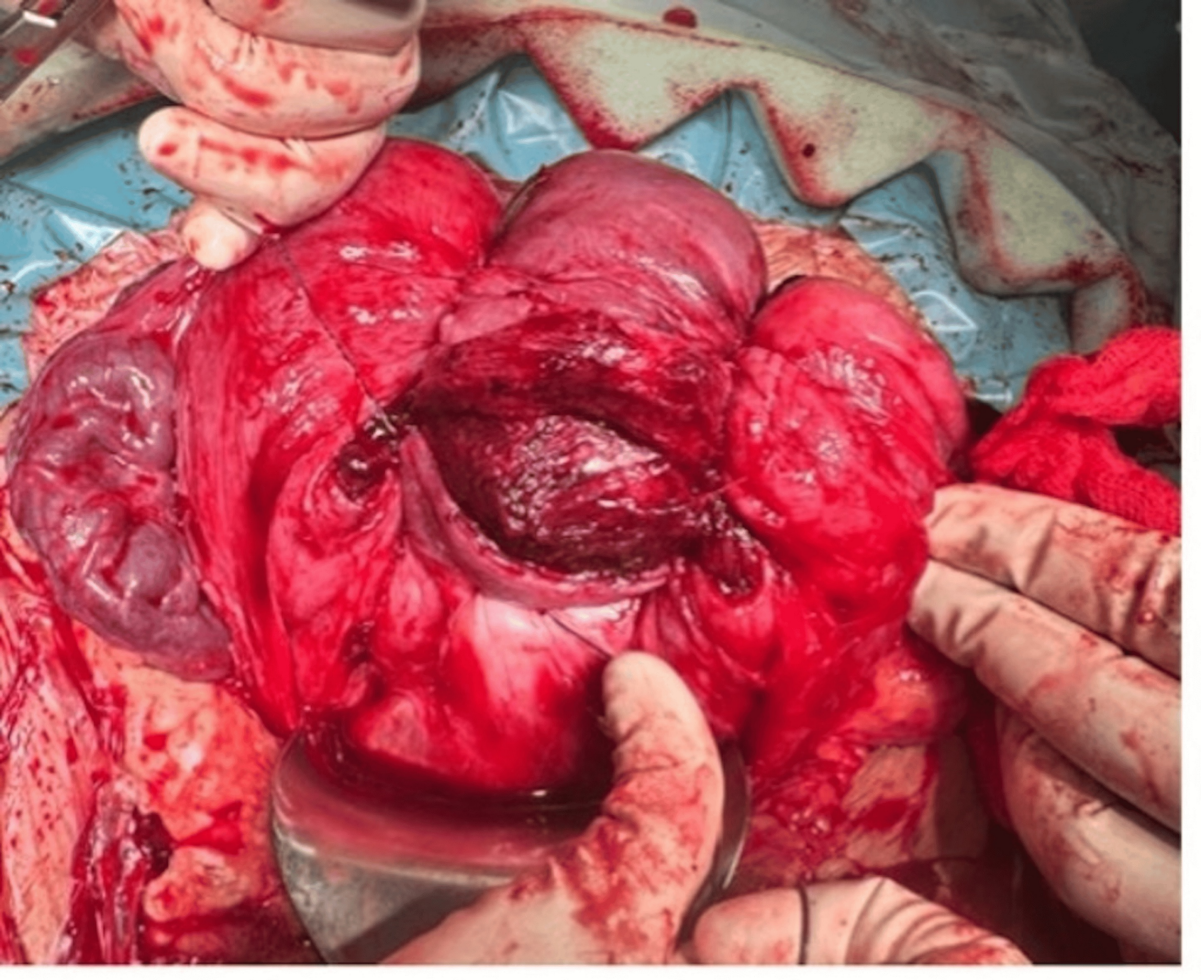
Tightening of circumferential cervical sutures for placenta accreta (Case 2).

**Figure 13:**
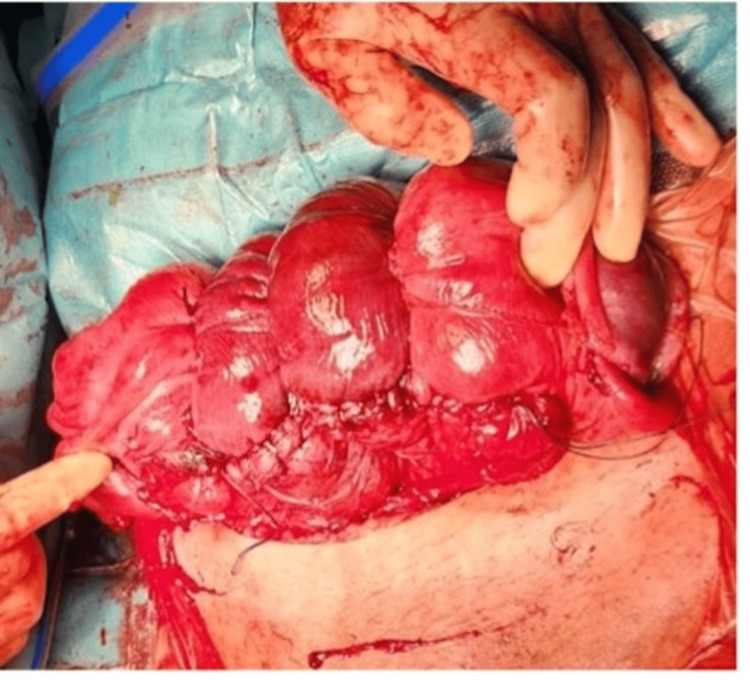
Well-compressed uterus with tightened ASSTT and circumferential cervical sutures (Case 2). ASSTT: Alternate Sequential Suture Tightening Technique

As an adjunct in placenta accreta spectrum, a circumferential cervical suture is applied. Using No. 1 Vicryl, the suture is passed below the cesarean incision and above the bladder reflection from the left anterior aspect to the posterior side, then from posterior to anterior on the right side at the same level before tightening (Case 2, Figures 11-13). This suture is intended to reduce bleeding from anastomosing vaginal arteries, which can contribute to significant hemorrhage in placenta accreta spectrum disorders. Postoperatively, patients were monitored for hemodynamic stability and postpartum bleeding. Follow-up was scheduled at seven, 14, 30, 90, and 180 days postoperatively and annually thereafter [13]. 

## Discussion

PPH remains a major cause of maternal morbidity and mortality worldwide [13,14]. It affects approximately 1-10% of deliveries, accounting for an estimated 14 million cases each year [2,15], and is associated with approximately 140,000 maternal deaths (about one death every four minutes) [16]. PAS occurs when a partial or complete absence of the decidua basalis allows chorionic villi to invade the myometrium. Depending on the depth of invasion, PAS is classified as placenta accreta, increta, or percreta [1]. Placenta percreta involves full-thickness myometrial invasion with possible extension to adjacent organs, such as the bladder or rectum [1,2]. The incidence of adherent placenta has been reported as approximately one in 588 deliveries [1]. Key risk factors include prior uterine injury (e.g., cesarean delivery, dilation and curettage, and other uterine surgery) and uterine infection [1,4,17-20]. PAS frequently coexists with placenta previa [1]. Among patients with placenta previa, the risk of PAS increases from 11% after one cesarean delivery to 60% after three or more cesarean deliveries [21,22].

Ultrasound is the primary imaging modality for PAS. Common sonographic findings include loss of the hypoechoic retroplacental zone, myometrial thinning, placental lacunae, subplacental hypervascularity, bridging vessels, and placental bulging [23]. Magnetic resonance imaging (MRI) may demonstrate uterine bulging, T2-weighted dark intraplacental bands, disruption of the uteroplacental interface, heterogeneous placental signal, and disorganized placental vascularity [24,25]. Management options for PAS include placental bed suturing, emergency hysterectomy, and conservative management with the placenta left in situ. Emergency hysterectomy can be technically challenging because of severe hemorrhage, poor tissue planes, the need for massive transfusion, uterine devascularization procedures, and postoperative intensive care unit (ICU) support. Delayed hysterectomy has been associated with higher rates of urinary tract infection and surgical site infection [26-28]. Conservative management may be complicated by endometritis (11%), ongoing bleeding, and readmission for exploratory surgery (29%) [29]. 

Management of PPH requires early recognition and rapid treatment of the underlying cause [13,30]. In the absence of PAS, most cases respond to first-line measures, including uterotonics, uterine massage, and balloon tamponade [13,30]. Ongoing bleeding may require operative management, most commonly uterine compression sutures or stepwise devascularization [13]. The B-Lynch suture was the first widely adopted uterine compression suture for severe atonic PPH. Although generally effective, it is technically demanding and requires reopening of the uterine cavity [13]. Reported complications include partial uterine wall necrosis or sloughing, cervical stenosis, and hematometra [13,31]. Multiple modifications have been described, including those by Hayman [7], Cho [8], Pereira [9], Hackethal [10], and Ouahba [11]. Despite these techniques, emergency hysterectomy is still required in some patients [4,5,10,11].

In 2015, a modified Hayman technique, the ASSTT (Ghosh suture), was reported in 12 patients with severe, refractory atonic PPH [13]. The mean operative time was four minutes (range, 2-7), the mean estimated blood loss was 1,625 mL (range, 1,300-1,900), and the mean length of stay was 10 days (range, 8-16) [13]. Hemorrhage was controlled with ASSTT alone in 11 patients (91.66%) [13]. No hysterectomy was required; one patient (8.33%) underwent additional bilateral internal iliac artery ligation [13]. Postoperative morbidity was limited to transient fever (n = 2) and mild superficial wound infection (n = 2), which resolved with conservative management [13]. At ≥2 years of follow-up, no infertility or menstrual disturbance was reported [13]. Persistent uterine atony may reflect inadequate uterine compression with standard modified B-Lynch techniques [13]. By providing more uniform compression, ASSTT may improve hemostasis and enhance hemorrhage control [13]. 

The corresponding author has used the ASSTT (Ghosh suture) in routine practice since 2009 and has applied it in more than 100 cases. Anxiety and fear after severe obstetric hemorrhage may reduce adherence to postpartum follow-up and may influence future reproductive planning. In the present report, two patients (one primigravida and one gravida 2) with a history of cesarean delivery were diagnosed with placenta accreta based on ultrasound and MRI. Both underwent an elective term cesarean delivery with prophylactic ASSTT and a circumferential cervical suture. The mean time required to place the Ghosh suture was four to five minutes, and the mean estimated blood loss was 450 mL (range, 450-500 mL). Neither patient required a blood transfusion, and both were discharged on postoperative day 3 without complications. Both reported regular menstrual cycles without sequelae during follow-up. The primigravida subsequently conceived spontaneously and underwent an uncomplicated elective-term repeat cesarean delivery three years later. The second patient underwent IVF for a third pregnancy (for sex selection) at 3.5 years. Antenatal imaging was unremarkable; however, intraoperatively, the placenta was again found to be morbidly adherent, and ASSTT with a circumferential cervical suture was reapplied. The patient was discharged on postoperative day 3 without complications. A physiologic rationale is proposed for the absence of uterine necrosis, hematometra, or adverse fertility outcomes with sequential tightening sutures. The uterus involutes rapidly immediately after delivery; therefore, after application of a compression suture, progressive reduction in uterine size may reduce the risk of sustained tissue ischemia. Using the circumference approximation (C = 2πr), a decrease in fundal height from 20 cm at delivery to 18 cm at two hours would correspond to a reduction in perimeter of more than 6 cm, which may relieve compressive tension. In addition, close apposition of the anterior and posterior uterine walls may limit intrauterine blood accumulation, thereby reducing the likelihood of hematometra. Finally, the sequential suturing pattern may provide more physiologic compression during normal postpartum involution without adversely affecting fertility [13].

## Conclusions

A standardized classification system for uterine compression suture techniques may improve procedural uniformity and support the development of evidence-based guidance across indications for PPH. These cases are frequently complex and may require advanced surgical expertise, multidisciplinary care, intensive care support, and timely access to blood products. Severe PPH remains a life-threatening obstetric emergency and a substantial clinical and medicolegal challenge because of its unpredictable course and outcomes. Timely recognition and referral of high-risk cases to tertiary centers or dedicated PPH services may reduce peripartum complications and maternal mortality. The Ghosh uterine compression suture appears to be a reliable uterus-sparing option for atonic PPH and placenta accreta spectrum disorders, particularly when combined with a circumferential cervical suture.
